# Determination of Lead, Cations, and Anions Concentration in Indoor and Outdoor Air at the Primary Schools in Kuala Lumpur

**DOI:** 10.1155/2014/408275

**Published:** 2014-07-22

**Authors:** Normah Awang, Farhana Jamaluddin

**Affiliations:** Environmental Health and Industrial Safety Programme, Faculty of Health Sciences, Universiti Kebangsaan Malaysia, Jalan Raja Muda Abdul Aziz, 50300 Kuala Lumpur, Malaysia

## Abstract

This study was carried out to determine the concentration of lead (Pb), anions, and cations at six primary schools located around Kuala Lumpur. Low volume sampler (MiniVol PM_10_) was used to collect the suspended particulates in indoor and outdoor air. Results showed that the concentration of Pb in indoor air was in the range of 5.18 ± 1.08 *μ*g/g–7.01 ± 0.08 *μ*g/g. All the concentrations of Pb in indoor air were higher than in outdoor air at all sampling stations. The concentrations of cations and anions were higher in outdoor air than in indoor air. The concentration of Ca^2+^ (39.51 ± 5.01 mg/g–65.13 ± 9.42 mg/g) was the highest because the cation existed naturally in soil dusts, while the concentrations of NO_3_^−^ and SO_4_^2−^ were higher in outdoor air because there were more sources of exposure for anions in outdoor air, such as highly congested traffic and motor vehicles emissions. In comparison, the concentration of NO_3_^−^ (29.72 ± 0.31 *μ*g/g–32.00 ± 0.75 *μ*g/g) was slightly higher than SO_4_^2−^. The concentrations of most of the parameters in this study, such as Mg^2+^, Ca^2+^, NO_3_^−^, SO_4_^2−^, and Pb^2+^, were higher in outdoor air than in indoor air at all sampling stations.

## 1. Introduction

Air pollution is generally the most widespread and obvious kind of environmental damage [1]. Kuala Lumpur, which is the federal capital and the largest city in Malaysia, is also suffering from air pollution problem. The last decade has seen its phenomenal growth as a centre of commerce in the region, and this trend is still continuing. With the increase in energy consumption and urbanization in Kuala Lumpur, the increase in ambient air pollution seems inevitable [2].

Air pollutants, which exist in the form of solid, semisolid, liquid, and gas, are emitted directly or indirectly from their sources. Some heavy metals such as lead and cadmium are common environmental pollutants in industrialised and developing countries [3]. Lead is a very toxic, nondegradable heavy metal that exists naturally in Earth's crust [4, 5]. Lead in the atmosphere arises from two major ways, which are primary sources including lead from mining activities and secondary sources such as industrial emission, battery manufacturing, and additives in motor vehicles gasoline. In any population, children are more vulnerable to lead exposure than adults because children have higher hand-to-mouth activities and higher rate of gastrointestinal absorption, and their developing brains are more sensitive to insults from lead exposure [6].

Ionic species either anions or cations can significantly be found in the form of particulate in the air especially during air pollution. Generally, anions such as sulphate (SO_4_^2−^) and nitrate (NO_3_^−^) are secondary particulates and usually dominate air pollution. Calcium (Ca^2+^) that arises from soils and magnesium (Mg^2+^) and chloride (Cl^−^) that comes from marine sources are some examples of cations [7].

This paper reports the concentration of lead, cations, and anions in air at the primary schools in Kuala Lumpur. Then, the concentrations of the parameters between indoor and outdoor air in every sampling station are compared.

## 2. Methodology 

### 2.1. Study Area

This study was conducted in Kuala Lumpur, which is a city characterised with highly congested traffic city. Six sampling points were chosen by using stratified random sampling, and size for known population was calculated.  Table 1 shows the list of the sampling stations.

### 2.2. Sampling and Analysis

Suspended particulate for indoor and outdoor air was sampled using low volume sampler (MiniVol PM_10_) for 24 h with fibre glass filter papers pore size 0.45 *μ*m and diameter 47 mm. The filter papers were then split into two parts. The first half was used for analysis of anions and cations, whilst the second half was used for analysis of lead. For lead concentration determination, a part of the filter paper was added with nitric acid and perchloric acid in the ratio of 16 : 4. Then, the mixture was filtered using vacuum pump with cellulose acetate filter papers (pore size 0.2 *μ*m), and 50 mL of deionised water was added. Lead concentration in the samples was determined using Inductively Coupled Plasma Mass Spectrophotometry (ICP-MS).

For anions and cations analysis, the second part of the filter paper was added with 40 mL of deionised water into centrifuge tube. Centrifugation was done for 45 min at 1500 rpm. Then, the mixture was filtered using vacuum pump and 100 mL of deionised water was added. All the samples were placed in polyethylene bottles at 4°C. The concentration of cations (Mg^2+^, Ca^2+^, and K^+^) was analysed using Atomic Absorption Spectroscopy, while the concentration of anions (NO_3_^−^ and SO_4_^2−^) was determined using HACH DR 2800 spectrophotometer. The data were then analysed using Statistical Package for the Social Sciences (SPSS) software version 16.

## 3. Results and Discussion 

### 3.1. Concentration of Lead for Indoor and Outdoor Air

Overall, this study indicated that lead concentration was higher for indoor air than outdoor air. For indoor air, the lead concentrations were in the range of 5.18 ± 1.08 *μ*g/g–7.01 ± 0.08 *μ*g/g. For outdoor air, the lead concentrations were in the range of 3.25 ± 0.35 *μ*g/g–4.09 ± 0.71 *μ*g/g (see Table 2). Based on one-way ANOVA test, there was a significant difference (*P* < 0.05) for the lead concentrations in indoor air. Station F showed the highest lead concentration for indoor air as the classroom was near the main road, which was highly congested during peak hours. Shen et al. [4] and Mohamed et al. [8] state that stationeries, desk paint, and coloured book wrappers contribute to lead in indoor air.

### 3.2. Concentration of Cations in Indoor and Outdoor Air

The concentration of Mg^2+^ in indoor air was in the range of 1.83–3.87 mg/g, while the concentration of Mg^2+^ in outdoor air was 2.63–4.41 mg/g (see Table 3). The concentration of Mg^2+^ was the lowest compared to other cations. Mg^2+^ is one of the components with high concentration in sea spray aerosol, which was far from sampling points. Next, for Ca^2+^, the range of concentration was quite high, 30.30 ± 2.09 mg/g–48.33 ± 1.97 mg/g in indoor air, while the range of concentration for outdoor air was 39.51 ± 5.01 mg/g–65.13 ± 9.42 mg/g (see Table 3).

This finding could be because the cation exists naturally in soils and Earth's crust; thus it has higher concentration in indoor air than outdoor air. Station A showed the highest concentration of Ca^2+^ in outdoor air. The indoor Ca^2+^ could probably come from soil dust introduced into the indoor environment. This finding was due to the construction project located in the school compound. There was a significant difference in Ca^2+^ concentrations for both indoor and outdoor air based on the statistical results. The concentration of K^+^ for indoor air was in the range of 38.07 ± 1.03–60.56 ± 3.71 mg/g, while the concentration of K^+^ for outdoor air was in the range of 37.64 ± 5.33 mg/g–66.33 ± 7.54 mg/g (see Table 3). Statistical results showed that the significant difference in the concentration of K^+^ was only in outdoor air. K^+^ is a mineral found in Earth's crust. The mineral is transferred from rocks to soil [9].

### 3.3. Concentration of Anions in Indoor and Outdoor Air

The concentration of SO_4_^2−^ in indoor air at every sampling point was in the range of 21.76 ± 1.13 *μ*g/g–23.42 ± 0.91 *μ*g/g, while the concentration of SO_4_^2−^ in outdoor air was in the range of 22.89 ± 2.04 *μ*g/g–23.95 ± 0.92 *μ*g/g (see Table 4). The concentration of ion SO_4_^2−^ was high in outdoor air due to the emission from motor vehicles in the heavy traffic nearby the schools. Emission of anthropogenic pollutants such as sulphur dioxide, vehicles exhaust, and natural emission from decaying plants and animals may increase the concentration of SO_4_^2−^ in the air [10]. The concentration of NO_3_^−^ in indoor air was in the range of 21.34 ± 0.97 *μ*g/g–24.80 ± 0.58 *μ*g/g, while the concentration of NO_3_^−^ in outdoor air was in the range of 29.72 ± 0.31 *μ*g/g–32.00 ± 0.75 *μ*g/g (see Table 4). Overall, the concentration of NO_3_^−^ was found higher in outdoor air than in indoor air. Fuels and exhaust emission from motor vehicles were the main sources for the anions in the air.

## 4. Conclusion 

Concentration of lead for both indoor and outdoor air at the sampling stations did not exceed the 1.5 *μ*g/m^3^ standard given by the Department of Environment. But the level of the contaminants must be monitored from time to time because exposure to the high concentration of lead can cause chronic health effects. All the sampling stations showed concentration of cations and anions higher in outdoor air than indoor air. This finding could be because the outdoor environment was exposed to higher sources of these cations and anions. Relative concentration for cations in this study was Ca^2+^ > K^+^ > Mg^2+^, while for anions it was NO_3_^−^ > SO_4_^2−^.

## Figures and Tables

**Table 1:** List of sampling stations.

Sampling station	Primary schools
A	SK Jalan Raja Muda Abdul Aziz
B	SK (L) Jalan Batu
C	SK Jalan Hang Tuah 1 dan 2
D	SK St. John 1 dan 2
E	SK (P) Pudu 1 dan 2
F	SK Convent Sentul 1 dan 2

**Table 2:** Concentration of Pb for both indoor and outdoor air according to sampling stations.

Sampling station	Concentration of Pb (*μ*g/g)	
	Indoor air	Outdoor air
A	6.05 ± 1.14	3.90 ± 1.04
B	6.01 ± 0.62	3.25 ± 0.35
C	7.01 ± 0.08	3.53 ± 0.05
D	5.64 ± 0.09	4.09 ± 0.71
E	5.18 ± 1.08	3.33 ± 0.08
F	8.15 ± 0.52	3.99 ± 1.15

**Table 3:** Concentration of Mg^2+^, Ca^2+^, and K^+^ for both indoor and outdoor air according to sampling stations.

Sampling station	Mg^2+^ (mg/g)		Ca^2+^ (mg/g)		K^+^ (mg/g)	
	Indoor	Outdoor	Indoor	Outdoor	Indoor	Outdoor
A	3.87 ± 0.05	4.41 ± 1.07	47.65 ± 2.12	65.13 ± 9.42	60.56 ± 3.71	61.69 ± 5.24
B	3.06 ± 0.18	3.28 ± 1.54	48.33 ± 1.97	61.49 ± 9.67	38.07 ± 1.03	37.64 ± 5.33
C	2.59 ± 0.99	3.23 ± 1.89	38.85 ± 0.72	56.03 ± 9.72	52.80 ± 1.41	66.33 ± 7.54
D	2.64 ± 0.87	3.03 ± 0.74	36.92 ± 1.56	52.34 ± 7.16	45.09 ± 1.09	64.25 ± 4.92
E	1.83 ± 0.39	2.81 ± 1.93	40.8 ± 0.53	60.56 ± 4.93	48.74 ± 1.34	50.16 ± 4.36
F	1.88 ± 0.11	2.63 ± 0.12	30.30 ± 2.09	39.51 ± 5.01	58.23 ± 0.97	49.28 ± 5.05

**Table 4:** Concentration of SO_4_^2−^ and NO^3−^ for both indoor and outdoor air according to sampling stations.

Sampling station	SO_4_^2−^ (mg/g)		NO^3−^ (mg/g)	
	Indoor	Outdoor	Indoor	Outdoor
A	21.76 ± 1.13	22.89 ± 2.04	25.36 ± 0.76	31.10 ± 0.08
B	22.81 ± 0.55	23.09 ± 1.35	23.17 ± 1.56	32.00 ± 0.75
C	23.42 ± 1.91	23.95 ± 2.51	21.79 ± 0.38	30.93 ± 0.41
D	23.05 ± 2.43	23.38 ± 2.74	22.53 ± 1.21	30.72 ± 0.26
E	22.76 ± 1.81	23.73 ± 1.21	24.80 ± 0.58	29.53 ± 0.63
F	22.71 ± 2.14	24.52 ± 1.03	21.34 ± 0.97	29.72 ± 0.31
